# Managing new premium-priced medicines in Europe

**DOI:** 10.1186/2052-3211-8-S1-K2

**Published:** 2015-10-05

**Authors:** Govin Permanand, Hanne Bak Pedersen

**Affiliations:** 1 World Health Organization (WHO) Regional Office for Europe Copenhagen, 2100, Denmark

## 

An issue of growing concern for health policy-makers in Europe is the continuing rise in spending on pharmaceuticals. In OECD countries pharmaceutical expenditure rose by 3.5% per year between 2000 and 2009, and averages 18-19% of total health expenditure [1]. Similar averages are seen across Europe, with pharmaceutical spending representing the largest component of ambulatory care [2]. Spending in some countries has dropped since 2009 on account of specific policy decisions taken due to the financial crisis, but in others this growth has remained constant [1,3]. Contributing to this is the continual introduction of new premium-priced medicines, particularly for biologicals given the appreciable number in development and their envisaged high prices [3-6]. And while the introduction of new therapies and current rapid pace of therapeutic innovation, particularly for noncommunicable diseases, is extremely positive from a patient perspective, managing their entry and longer term affordability especially under health insurance schemes and vis-à-vis existing lower-cost therapies poses a series of challenges to policy-makers regarding therapeutic complexity and higher costs [7].

To mitigate such pressures and to balance the demand for new medicines and the financial impact of their introduction, further development of systems and processes to optimize the entry of new medicines is necessary across Europe; this applies to countries with well-developed medicine policies and regulation traditions and those with less mature systems. And while many European countries have not traditionally required active priority-setting for access to medicines, appraising new medicines using pharmacoeconomics is increasingly seen as critical to improve efficient spending while maintaining an appropriate balance between access and cost-effectiveness. Indeed, policy-makers are in need of wider guidance on how to optimise the entry of new medicines to ensure the financial sustainability of their health care systems while encouraging the development of new treatments to address areas of unmet clinical need.

Although not an exhaustive list, areas in which the challenges around the sustainable management of new medicines are especially acute include:

• new medicines for patients with cancer where the price of new drugs has doubled over the past 10 years, up to US$10,000 per month, and often with little relationship between reimbursed costs and associated health benefits [5,8]

• new therapeutics for hepatitis C where patients are potentially being denied new effective direct-acting antivirals due to extremely high prices [9,10]

• orphan drugs where there is considerable unmet need for small patient populations, and where annual acquisition costs can be as high as US$500,000 per patient per year [11,12].

These represent examples of new premium-priced medicines which carry considerable implications for countries' health budgets due to being either high volume (for treating many patients) or high cost (because of the cost of a single course of treatment).

The importance of this issue, and the need for guidance across European countries, is underscored by the results of a 2014 query undertaken by the Pharmaceutical Pricing and Reimbursement Information (PPRI) Network (i.e. a network of competent authorities hosted by Gesundheit Österreich GmbH (GÖG), a WHO Collaborating Centre for Pharmaceutical Pricing and Reimbursement Policies based in Vienna, Austria, see E4). The survey of 42 countries, most of which are in Europe, revealed that countries are struggling with the overall issue of defining what constitutes a high-cost or premium-priced medicine [[7], annex 1]. Additionally, countries' understandings of a threshold for what constitutes an innovative advance over existing (lower-cost) therapies were shown to differ, with respondents noting that their countries did not have specific policies for the pricing and reimbursement of premium-priced medicines versus other medicines (although several are working on inpatient policies in particular).

Building on the PPRI query, and with the aim to help facilitate debate on policies around the introduction of new high-cost or premium-priced medicines in Europe, the World Health Organization Regional Office for Europe (WHO Europe), working with a number of partners, undertook a review of the specific policies and principles for managing their entry (including financing) across the different phases of the medicine product cycle (note that for the purposes of the WHO report, a broad definition of a new premium-priced medicine as one whose acquisition cost is greater than EUR 10,000 per patient per year for the public payer, and is replacing an existing medicine (also covered by the public payer), was adopted). Figure 1 locates potential policy actions throughout the medicines continuum; that is, from pre- to peri- to post-launch activities. This serves to indicate where the value of individual patient health outcomes from medicines treatment may be considered.

**Figure 1:**
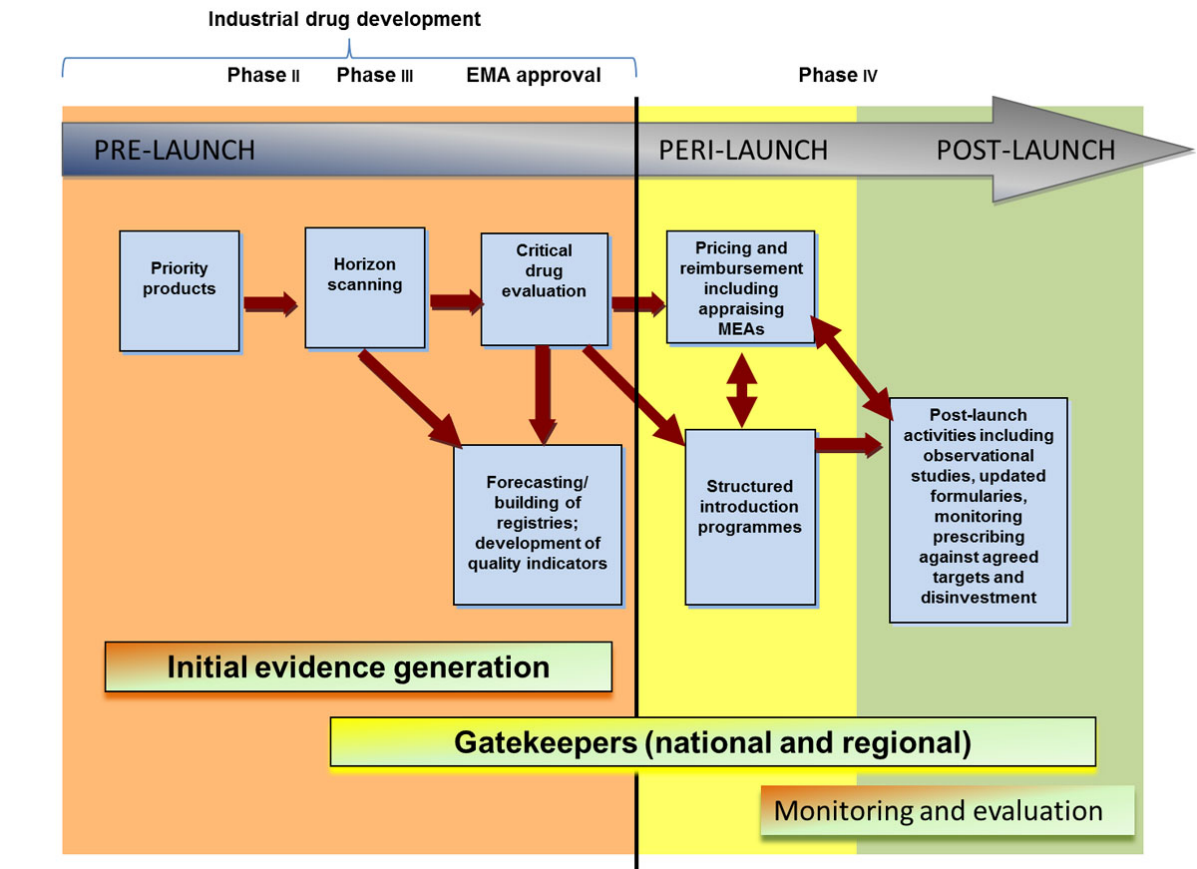
Activities to manage the entry of new medicines Source: [7] (adapted from [10,13,14]

Pre-launch activities provide policy-makers with a forward-looking perspective on new medicines in development. They can systematically anticipate and prioritise therapeutic innovation with the highest potential for impact on potential clinical and treatment outcomes, and health system impact (cost and benefit to patients and budget implications). Peri-launch activities address, among other things, issues of access and affordability and are generally around pricing and reimbursement policies, with the aim of ensuring that prices reflect clinical and therapeutic value for the patient. The use of health technology assessment (HTA) is also crucial here. Post-launch activities are those undertaken to address the appropriate and sustainable use of medicines, and oriented around an evidence-based assessment of their risk-benefit profile over time.

The WHO Europe report makes a series of suggestions on potential policy choices/directions, for policy-makers in Europe to consider, and these are oriented around the three phases indicated in Figure 1. Key steps in these processes should include methods to distinguish and reward meaningful clinical innovation, as well as evaluation mechanisms to assess the benefits in practice of the introduction of the medicines and impacts on health system budgets. Overall, amongst its recommendations for certain policy directions, the report concludes that:

• Prioritization processes should incorporate principles of collaboration and transparency, as a lack of collaborative and transparent policy-making and prioritization runs the risk of unfair and arbitrary treatment decisions and inefficient systems.

• Cooperation between stakeholders needs to involve better balancing of the value of innovation with equitable, affordable patient access. For while industry needs to be rewarded for its research and development efforts and the risk companies assume in pursuing innovation, it is also important to ensure that countries do not have to limit access because they cannot afford new medicines that represent a true therapeutic advance.

• In view of the considerable costs involved in these areas, collaboration among regional or subregional health systems could benefit from including a particular focus on chronic care, specialty medicines and rare diseases, such that networks of information exchange for new priority medicines in Europe including pricing trends, treatment protocols and guidelines, common principles for the registries for patient characterization and effectiveness and similar can offer a way forward [7].

It is clear that decision-makers across Europe will increasingly be faced with difficult choices in respect of new pharmaceuticals, and that achieving a sustainable balance between ensuring access and affordability around genuine therapeutic advances and treatment outcomes will be paramount.

## References

[B1] Health OECD data [online database] 2014 Paris: OECD Publishing (http://data.oecd.org/health.htm, accessed 10 August 2015)

[B2] Godman B Wettermark B Hoffmann M Andersson K Haycox A Gustafsson L Multifaceted national and regional drug reforms and initiatives in ambulatory care in Sweden: global relevance Expert Rev Pharmacoecon Outcomes Res 2009 9 1 65 83 10.1586/14737167.9.1.65 19371180

[B3] Pathways to value: pharma in a changing world 2013 Oxford: Meteos (PharmaFutures Global Conclusions, No. 5; http://apps.who.int/medicinedocs/en/d/Js20202en/, accessed 10 August 2015)

[B4] Shaw J Sicree R Zimmet P Global estimates of the prevalence of diabetes for 2010 and 2030 Diabetes Res Clin Pract 2010 87 1 4 14 10.1016/j.diabres.2009.10.007 19896746

[B5] Abboud C Berman E Cohen A Cortes J De Angelo D Deininger M The price of drugs for chronic myeloid leukemia (CML) is a reflection of the unsustainable prices of cancer drugs: from the perspective of a large group of CML experts Blood 2013 121 22 4439 4442 23620577 10.1182/blood-2013-03-490003 PMC4190613

[B6] Kaiser J Personalized medicine: new cystic fibrosis drug offers hope, at a price Science 2012 335 6069 645 10.1126/science.335.6069.645 22323790

[B7] WHO Europe ‘Access to new medicines in Europe: technical review of policy initiatives and opportunities for research’ 2015 WHO: Copenhagen available at: http://www.euro.who.int/en/health-topics/Health-systems/medicines/publications2/2015/access-to-new-medicines-in-europe-technical-review-of-policy-initiatives-and-opportunities-for-collaboration-and-research

[B8] Kelly R Smith T Delivering maximum clinical benefit at an affordable price: engaging stakeholders in cancer care Lancet Oncol 2014 15 3 e112 e118 10.1016/S1470-2045(13)70578-3 24534294

[B9] Sofosbuvir 400mg tablet (Sovaldi®) SMC advice [website] 2014 Glasgow: Scottish Medicines Consortium http://www.scottishmedicines.org.uk/SMC_Advice/Advice/964_14_sofosbuvir_Sovaldi/sofosbuvir_Sovaldi

[B10] Malmström R Godman B Diogene E Baumgärtel C Bennie M Bishop I Dabigatran – a case history demonstrating the need for comprehensive approaches to optimize the use of new drugs Front Pharmacol 2013 4 39 23717279 10.3389/fphar.2013.00039 PMC3653065

[B11] Cohen P Felix A Are payers treating orphan drugs differently? Journal of Market Access & Health Policy 2014 2 23513 10.3402/jmahp.v2.23513 PMC4865792 27226840

[B12] Michel M Toumi M Access to orphan drugs in Europe: current and future issues Expert Rev Pharmacoecon Outcomes Res 2012 12 1 23 29 10.1586/erp.11.95 22280193

[B13] Wettermark B Godman B Eriksson C van Ganse E Garattini S Joppi R Einführung neuer Arzneimittel in europäische Gesundheitssysteme [Introduction of new medicines into European healthcare systems] GGW 2010 10 3 24 34

[B14] Godman B Malmström R Diogene E Gray A Jayathissa S Timoney A Are new models needed to optimize the utilization of new medicines to sustain healthcare systems? Expert Rev Clin Pharmacol 2015 8 1 77 94 10.1586/17512433.2015.990380 25487078

